# Role of PCSK9 in Homocysteine-Accelerated Lipid Accumulation in Macrophages and Atherosclerosis in ApoE^−/−^ Mice

**DOI:** 10.3389/fcvm.2021.746989

**Published:** 2021-10-01

**Authors:** Ping Jin, Dengfeng Gao, Guangzhi Cong, Ru Yan, Shaobin Jia

**Affiliations:** ^1^Department of Cardiology, The Second Affiliated Hospital of Xi'an Jiaotong University, Xi'an, China; ^2^Heart Center and Cardiovascular Institute, General Hospital of Ningxia Medical University, Yinchuan, China

**Keywords:** PCSK9, homocysteine, macrophages, cholesterol efflux, atherosclerosis

## Abstract

**Background:** Homocysteine (Hcy) has been established as an independent risk factor for atherosclerosis, and the involvement of hyperhomocysteinemia (HHcy) in atherosclerotic lesions is complex. Proprotein convertase subtilisin kexin 9 (PCSK9) has vital importance in lipid metabolism, and its inhibitors have intense lipid-lowering and anti-atherosclerotic effects. However, the underlying effect of PCSK9 on HHcy-accelerated dyslipidemia of macrophages is still uncertain. The purpose of this study was to investigate the potential role of PCSK9 in Hcy-induced lipid accumulation and atherosclerotic lesions.

**Methods:** *In vitro*, gene and protein expressions were assessed by real-time quantitative PCR and western blot in THP-1 macrophages with Hcy incubation. Lipid accumulation and cholesterol efflux were evaluated with Hcy treatment. SBC-115076 was used to examine the role of PCSK9 in ATP-binding cassette transporter A1 and G1 (ABCA1 and ABCG1)-dependent cholesterol efflux. *In vivo*, lesion area, lipid deposition and collagen contents were determined in aortas of ApoE^−/−^ mice under a methionine diet. SBC-115076 was subcutaneously injected to explore the potential effects of PCSK9 inhibition on alleviating the severity of HHcy-related atherosclerotic lesions.

**Results:** In THP-1 macrophages, Hcy dose- and time-dependently promoted PCSK9 gene and protein levels without regulating the translation of Low-density lipoprotein receptor (LDLR). SBC-115076 used to inhibit PCSK9 largely alleviated lipid accumulation and reversed the cholesterol efflux to apolipoprotein-I(apoA-I) and high-density lipoprotein (HDL) mediated by ABCA1 and ABCG1. In ApoE^−/−^ mice, methionine diet induced HHcy caused larger lesion area and more lipid accumulation in aortic roots. SBC-115076 reduced atherosclerotic severity by reducing the lesion area and lipid accumulation and increasing expressions of ABCA1 and ABCG1 in macrophages from atherosclerotic plaque. In addition, SBC-115076 decreased plasma Hcy level and lipid profiles significantly.

**Conclusion:** PCSK9 promoted lipid accumulation via inhibiting cholesterol efflux mediated by ABCA1 and ABCG1 from macrophages and accelerated atherosclerotic lesions under HHcy treatment. Inhibiting PCSK9 may have anti-atherogenic properties in HHcy-accelerated atherosclerosis.

## Introduction

Atherosclerosis, chronic inflammation of arteries with lipid accumulation and plaque formation, is the basic pathophysiology of atherosclerotic cardiovascular disease (ASCVD) (1). Elevated plasma low-density lipoprotein cholesterol (LDL-C) is the most important risk factor for ASCVD, and the accumulation of modified LDL-C in macrophages and the formation of foam cells are characteristic changes of atherosclerosis (2). Imbalance of cholesterol intake, esterification and efflux, inflammation and apoptosis of macrophages jointly promote the formation and rupture of unstable plaques and lead to the occurrence of serious clinical events such as acute myocardial infarction and stroke. ATP-binding cassette transporter A1 and G1 (ABCA1 and ABCG1) are important members of ABC family and maintain cholesterol balance by being responsible for cholesterol efflux (3).

Homocysteine (Hcy) is an intermediate metabolite of methionine *in vivo* (4–6). Its mechanism of promoting atherosclerosis is complex and has not been clarified. ASCVD patients with hyperhomocysteinemia (HHcy) have more unstable plaque, more complex coronary atherosclerotic lesions and worse prognosis than those without HHcy (7–9). Liver X receptor alpha (LXRα) is a key regulator of ABCA1 and ABCG1, and our previous work (10) showed that HHcy accelerated lipid accumulation and atherosclerotic lesions by inhibiting LXRα-ABCA1 and ABCG1 pathway mediated cholesterol efflux. However, the improvement with treatment with the LXRα agonist T0901317 was limited, and the impact on lipid profiles was negligible. These results suggest that the molecular mechanism of Hcy induced lipid accumulation in macrophages needs further elucidation.

Proprotein convertase subtilisin kexin 9 (PCSK9) was discovered in 2003 (11). It is closely involved in lipid metabolism and atherosclerosis. In recent years, PCSK9 has attracted much attention in the field of lipid-lowering because of the powerful reduction of plasma LDL-C levels, amelioration of main adverse cardiovascular events and effect on stabilizing plaques with its inhibition (12, 13). In addition to promoting the lysosomal degradation of LDL receptor LDLR on the surface of hepatocytes (14), PCSK9 can also act directly on blood vessel walls, by disrupting lipid intake and efflux of macrophages, promoting the secretion of inflammatory factors and inducing apoptosis to participate in atherosclerosis and even plaque destabilization (15, 16). Ding et al. (17) found that silencing PCSK9 in THP-1 macrophages could inhibit the formation of foam cells by downregulating the scavenger receptor CD36. Adorni et al. (18) found that PCSK9 could directly inhibit the mRNA and protein levels of ABCA1 to suppress cholesterol efflux in mouse peritoneal macrophages and promote the formation of foam cells. This evidence indicates that PCSK9 might be a key regulator in lipid metabolism of macrophages, but the effect of PCSK9 on HHcy-induced dyslipidemia is rarely reported.

The purpose of the present study was to investigate the potential role of PCSK9 in Hcy-induced lipid accumulation and atherosclerotic lesions and also provide more evidence of PCSK9 inhibition for ASCVD patients with HHcy.

## Materials and Methods

### Materials and Regents

RPMI 1640 medium and fetal bovine serum (FBS) were purchased from Gibco (New York, NY, USA). Hcy, phorbol-12-myristate-13-acetate (PMA) and Oil-red O dye were supplied from Sigma-Aldrich (St. Louis, MO, USA). SBC-115076 and T0901317 was purchased from Selleck Chemicals (Houston, TX, USA). PCSK9 antibody was obtained from Cell Signaling Technology (Danvers, MA, USA). LDLR, ABCG1, CD68, β-actin and horseradish peroxidase (HRP) goat-anti-rabbit antibodies were both obtained from Abcam (Cambridge, MA, USA). ABCA1 antibody was purchased from Novus Biologicals (Littleton, CO, USA). LXRα was purchased from Santa Cruz Biotechnology (Santa Cruz, CA, USA). 22- [- (7- nitrobenz- 2- oxa- 1,3- diazol- 4- yl) amino]- 23, 24- bisnor- 5- cholen- 3β- ol (22-NBD cholesterol) was obtained from Invitrogen (Grand Island, NY, USA). PCR primers were supplied from Sangon Biotech (Shanghai). ApoA-I was supplied from Calbiochem (San Diego, CA, USA) and oxidized-LDL and high-density lipoprotein (HDL) were both obtained from Xiesheng Biotechnology (Beijing).

### THP-1 Monocyte-Derived Macrophages Cell Culture

THP-1 human monocytic leukemia cells were obtained from the American Type Culture Collection (Rockville, MD, USA) and were maintained in RPMI 1640 supplemented with 10% FBS at 37°C in a humidified atmosphere of 5% CO_2_. Differentiation of THP-1 cells into macrophages was induced by using 100 nmol/L PMA for 24 h until adherent to the cell culture plate. To mimic the foam cell formation, we replaced the medium with 50 μg/mL ox-LDL in a serum-free medium for 24 h to fully differentiate THP-1 macrophages to foam cells. As described previously (10), we treated THP-1 macrophages with various concentration of Hcy (50, 100, and 200 μmol/L) or vehicle (equivalent volume of PBS) for 24 or incubated with 100 μmol/L Hcy under different exposure times (0, 6, 12, 24, and 48h). The concentration of Hcy was based on the previous studies (19–21). To explore the potential role of PCSK9, we first incubated THP-1 cells with different concentrations of SBC-115076 (5, 10, and 20 μmol/L) for 24 h to inhibit PCSK9 expression. Then we treated THP-1 cells together with Hcy (100 μmol/L) and SBC-115076 (20 μmol/L) for 24 h. We also incubated THP-1 macrophages with Hcy (100 μmol/L) and T0901317 (5 μg/mL) for 24 h. All cell experiments were based on a cell viability of 90% or higher. Each independent experiment was performed at least three times.

### Quantitative PCR Analysis

THP-1 macrophages (51 × 0^5^ cells) were plated on 12-well plates, then washed twice with PBS after incubation for 24 h. Total cellular RNA was extracted by using the RNAprep Pure cell/Bacteria Kit (TIANGEN, China) following the manufacturer's instructions. Reverse transcription involved using the PrimeScript Master Mix kit (Takara, Tokyo, Japan). The cDNA of PCSK9 and LDLR were anlayzed by real-time quantitative PCR on iQ5 Multicolour RT-PCR Detection System (Bio-rad, Hercules, CA, USA) with SYBR Select Master Mix (Invitrogen, Carlsbad, CA, USA). GAPDH was selected as the housekeeping gene. Primers for the genes were used in this study: homo PCSK9, forward: CCCAGGTCTGGAATGCAAAG and reverse: CTGCCTGTAGTGCTGACGT; homo LDLR, forward: TGGACATCTACTCGCTGGTG and reverse: TCGATGCTTGAGATGGAGTG; homo GAPDH, forward: CTGACTTCAACAGCGACACC and reverse: TGCTGTAGCCAAATTCGTTG.

### Immunoblotting

Cultured cells were lysed with RIPA buffer (Genshare, Shaanxi, China). Protein concentration was determined by BCA protein assay reagent kit (Thermo Fisher Scientific). 30 μg of total protein samples were separated on 10% SDS-PAGE and transferred to a PVDF membrane (Roche Diagnostics, Minneapolis, MN). After blocking with 5% nonfat milk in TBST for 1 h at room temperature, the PVDF membranes were subsequently incubated with anti-PCSK9, anti-LDLR, anti-LXRα, anti-ABCA1, anti-ABCG1, or anti-β-actin antibodies at 4°C overnight, then HRP-conjugated secondary antibodies with primary antibodies for 1 h at room temperature. Immunoreactive bands was visualized by the ECL test.

### Oil-Red O Staining

At the end of the intervention, cells were fixed with 4% paraformaldehyde for 30 min and then stained with 0.5% Oil-red O for 15 min at 37°Cto identify lipid droplets in cytoplasm, followed by nuclear counterstaining with hematoxylin for 5 s. The Oil-red O positive area meant the percentage of red area and total area of THP-1 macrophages.

### Cholesterol Efflux Assay

Cholesterol efflux was determined as previously described (22). After treating with 100 μmol/L Hcy for 18 h and then with 1 μg/mL 22-NBD cholesterol for additional 6h, THP-1 cells were incubated in RPMI1640 with 0.2% bovine serum albumin containing 15 μg/mL apoA-I or 50 μg/mL HDL for 6 h. Subsequently, the supernatant was collected and the fluorescence-labeled cholesterol released from cells into the medium was measured. The percent of cholesterol efflux was calculated by the following equation: [total medium counts/(total cellular counts + total medium counts)] × 100%. The specific efflux to apoA-I or HDL was calculated by the following equation:(efflux to apoA-I or HDL-non-specific efflux to RPMI 1640).

### Animals and Experimental Procedures

The experimental protocols were in accordance with the principles of animal welfare and were approved by the Institutional Ethics Committee for Animal Experiments of Ningxia Medical University. We purchased 36 male 6-week-old ApoE^−/−^ mice from the Animal Center of Peking University Health Science Center (Beijing). All mice were housed in a controlled environment (252 ± °C, 12-h light-dark cycles) with a standard chow diet and free access to water. Mice were equally divided into three groups for treatment: control group (AC group, *n* = 12), methionine-supplemented group (AHM group, *n* = 12) and methionine-supplemented+ SBC-115076 group (AHS group, *n* = 12). According to the sample size selection in previous experiments, we set a sample size of 12 mice in each group (21, 23, 24). The mice in AC group were fed with a standard rodent maintenance diet as recommended by the American Institute of Nutrition-93 purified diet (AIN-93G); while mice in the other two groups were fed with a control diet plus 17 g/kg methionine food with or without 8 mg/kg SBC-115076 subcutaneous injected twice a week from week 8.

### Detection of Plasma Levels of Hcy and Lipids

At the end of the intervention, all the mice were fasting and deprived of water overnight, and 1% pentobarbital sodium was intraperitoneally injected (50 mg/kg). After anesthesia, blood samples from orbital vein were collected and centrifuged immediately. The levels of plasma Hcy, total cholesterol (TC), triglycerides (TG), LDL-C and HDL-C were detected by automatic biochemical instrument (Siemens, Germany).

### Hematoxylin(HE), Oil-Red O and Masson Staining for Atherosclerotic Lesions Analysis

Cross-sectional analysis at the aortic sinus with 10 μm thickness were used to evaluate the severity of atherosclerotic lesions. Hematoxylin (HE) staining was performed to evaluate the lesion area; Oil-red O (ORO) staining was performed to evaluate the lipid accumulation and Masson trichrome staining was performed to evaluate the collagen deposition. The percentage of positive staining area was calculated respectively.

### Immunofluorescence Analysis

To evaluate the effects of HHcy and SBC-115076 on the expression of PCSK9, ABCA1 and ABCG1 in macrophages of atherosclerotic lesions, sections of plaque areas were prepared. CD68^+^PCSK9^+^, CD68^+^ABCA1^+^ and CD68^+^ABCG1^+^ cells were analyzed by double immunofluorescence staining with primary antibodies for mouse CD68 or rabbit PCSK9, ABCA1 and ABCG1 overnight at 4°C. Then with specific fluorescent labeled secondary antibodies at 37°C for 1 h. All images were quantified by using Image-Pro Plus 6.0.

### Statistical Analysis

All data are expressed as the mean ± standard error of the mean (SEM). Intergroup differences were analyzed by Student *t*-test comparison of two groups. One-way ANOVA was used to compare three groups. *P* < 0.05 was considered statistically significant.

## Results

### Hcy Increased PCSK9 Expression in THP-1 Macrophages

**Figure 1:**
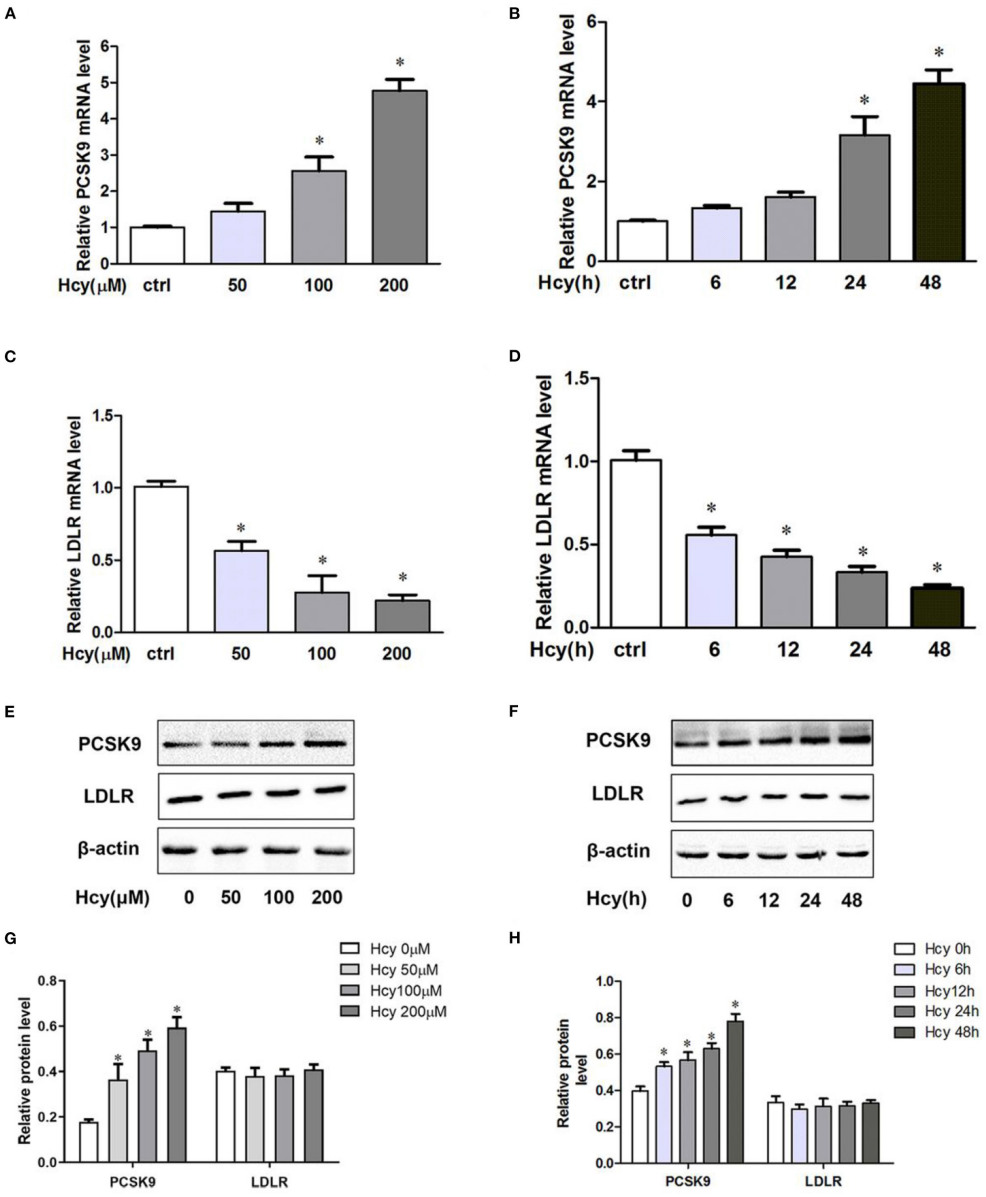
Homocysteine(Hcy) increased proprotein convertase subtilisin kexin 9(PCSK9) level in THP-1 macrophages, and SBC-115076 reversed this effect. THP-1 macrophages were treated different concentrations of Hcy (50, 100 and 200 μmol/L) **(A,C)** or vehicle (equivalent volume of PBS) for 24 h or incubated with 100 μmol/L Hcy under different exposure times (0, 6, 12, 24, and 48 h) **(B,D)**. The mRNA levels of PCSK9 and LDLR were analyzed by real-time quantitative PCR. The protein levels of PCSK9 and LDLR were measured by western blot analysis **(E–H)**. Data represent mean ± standard error of the mean (SEM). Each experiment was performed three times. ^*^p < 0.05 vs. the control group (Hcy 0 μmol/L, which refers to incubation with PBS only). Hcy, homocysteine.

To shed light on the effect of Hcy on PCSK9 and LDLR in THP-1 macrophages, we investigated the mRNA and protein levels of PCSK9 and LDLR. Hcy dose- and time-dependently increased PCSK9 level at both the mRNA (Figures 1A,B) and protein levels (Figures 1E–H). When incubating THP-1 macrophages with 100 μmol/L Hcy for 24 h, PCSK9 mRNA and protein levels increased about 168 and 182%, respectively. Hcy decreased the mRNA level of LDLR (Figures 1C,D) but had little effect on its protein level (Figures 1E–H).

### SBC-115076 Reversed Hcy-Upregulated PCSK9

**Figure 2:**
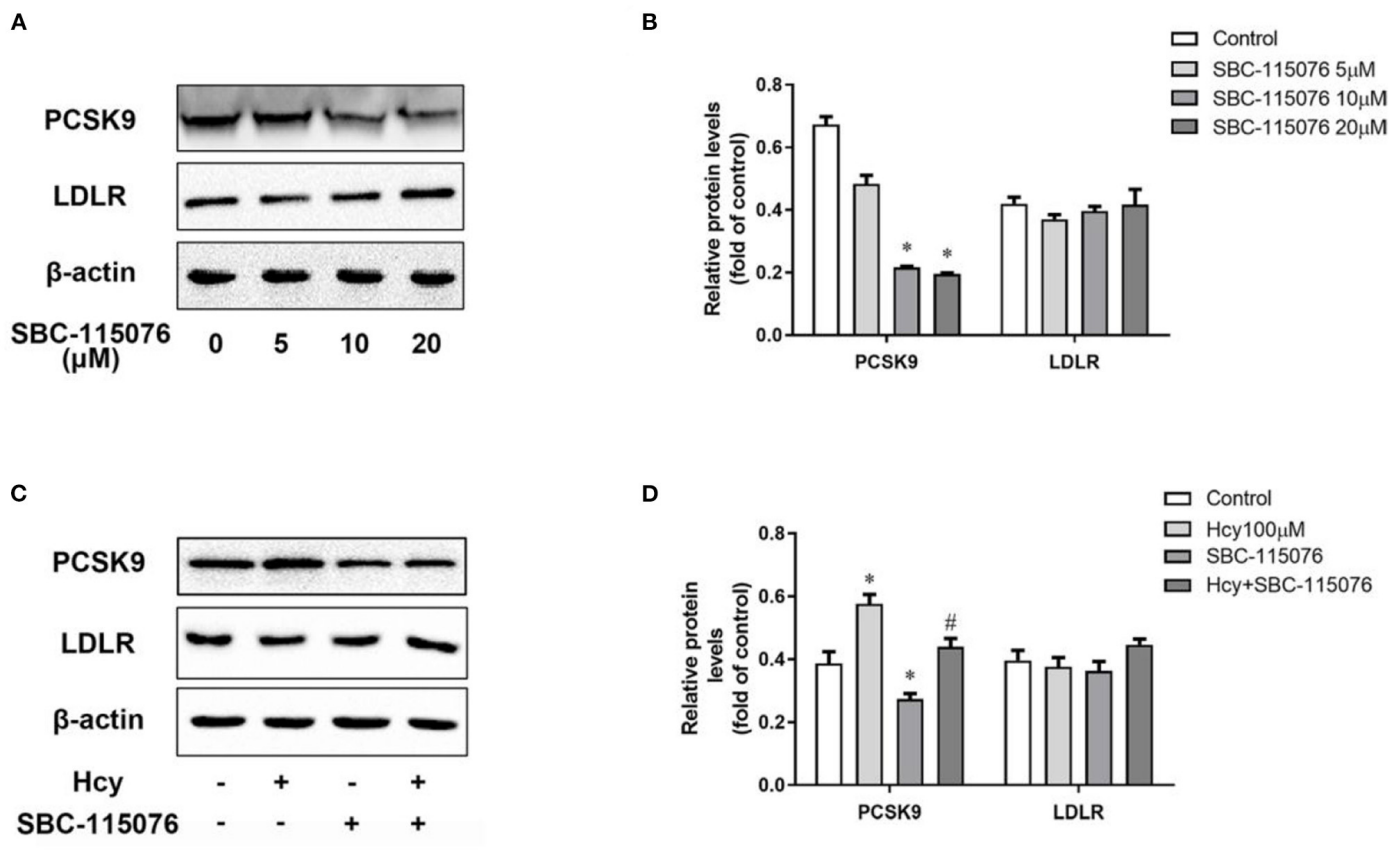
SBC-115076 reversed the up-regulation of PCSK9 caused by Hcy. Protein levels of PCSK9 and LDLR were determined by western blot analysis with 5, 10, and 20 μmol/L SBC-115076 for 24 h **(A,B)** in THP-1 macrophages. Then cells were co-incubated with 100 μmol/L Hcy and 20 μmol/L SBC-115076 for 24 h. Protein levels of PCSK9 and LDLR were determined **(C,D)**. Data represent mean ± standard error of the mean (SEM). Each experiment was performed three times. *p < 0.05 vs. the control group(Hcy 0 μmol/L, which refers to incubation with PBS only). ^#^*p* < 0.05 vs. Hcy group(Hcy 100 μmol/L). Hcy, homocysteine.

We used SBC-115076, an antagonist of PCSK9, to explore the effect of Hcy on PCSK9 and LDLR protein levels. SBC-115076 dose-dependently suppressed the protein expression of PCSK9 in THP-1 macrophages, but had little effect on LDLR protein level (Figures 2A,B). On co-incubation with Hcy and SBC-115076, SBC-115076 decreased the protein level of PCSK9 about 38% as compared with Hcy treatment (Figures 2C,D). Thus, Hcy promoted PCSK9 expression, and SBC-115076 reversing the effects, which may not be *via* the regular PCSK9/LDLR lysosome degradation pathway.

### SBC-115076 Reversed the Hcy-Downregulated ABCA1 and ABCG1 Levels, Lipid Accumulation and Inhibition of Cholesterol Efflux

**Figure 3:**
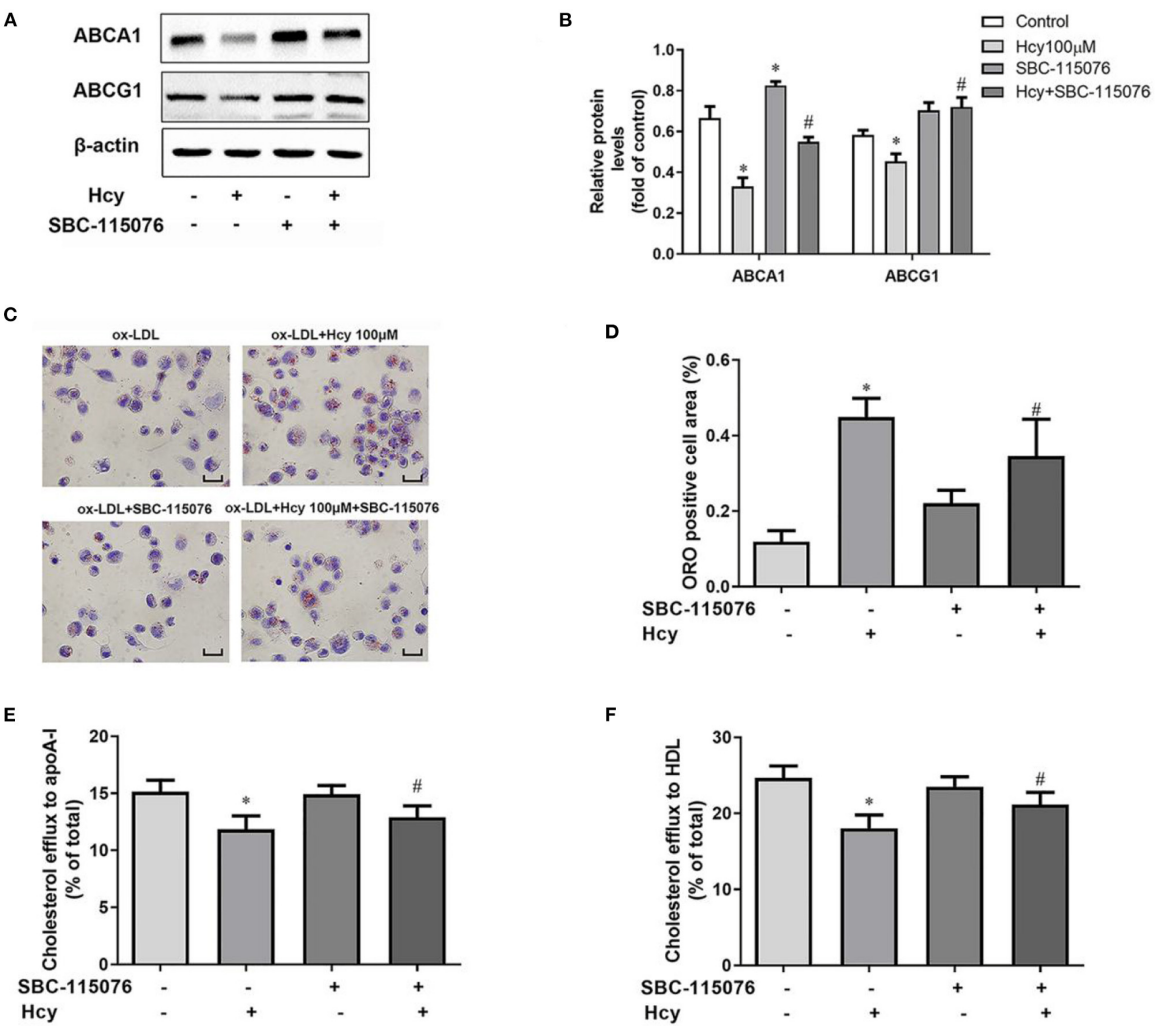
SBC-115076 reversed the downregulation of ABCA1 and ABCG1, lipid accumulation and inhibition of cholesterol efflux induced by Hcy. THP-1 macrophages were treated with 100 μmol/L Hcy and 20 μmol/L SBC-115076 for 24 h. The protein expressions of ABCA1 and ABCG1 were determined by western blot analysis **(A,B)**. Oil-red O (ORO) staining was used to assess lipid accumulation **(C,D)**. Cholesterol efflux to apoA-I or HDL from THP-1 macrophage-derived foam cells was assessed **(E,F)**. Data represent mean ± standard error of the mean (SEM). Each experiment was performed three times. *p < 0.05 vs. the control group (Hcy 0 μmol/L, which refers to incubation with PBS only). ^#^*p* < 0.05 vs. Hcy group (Hcy 100 μmol/L). Scale bar = 25 μm. Hcy, homocysteine.

In our previous study, we found (10) that Hcy promoted accumulation of lipid by inhibiting cholesterol efflux mediated by LXRα-ABCA1/ABCG1 pathway in THP-1 macrophages. ABCA1 and ABCG1 are the key regulators of the cholesterol efflux to lipid-free apoA-I and HDL, respectively. Hence, we co-incubated THP-1 macrophages with Hcy and SBC-115076 to explore the role of PCSK9 in Hcy-accelerated lipid accumulation in macrophages. Firstly, we assessed protein levels in THP-1 macrophages. Hcy decreased the protein levels of LXRα, ABCA1 and ABCG1, but SBC-115076 increased the protein levels as compared with Hcy treatment (Figures 3A,B). However, SBC-115076 had no effect on the protein expression of LXRα with or without Hcy incubation (Supplementary Figures 1A–D). Secondly, we assessed lipid accumulation under Hcy and SBC-115076 treatment by using Oil-red O staining. Hcy significantly promoted lipid accumulation, and SBC-115076 alleviated the lipid accumulation by about 23% as compared with treatment (Figures 3C,D). Then we incubated cells with 100 μmol/L Hcy and 5 μg/mL T0901317 for 24 h and the data showed that PCSK9 protein level was also decreased by T0901317 (Supplementary Figures 1E,F). Thirdly, we used 22-NBD cholesterol to assess cholesterol efflux. Inhibition of cholesterol efflux to apoA-I and HDL by Hcy was moderately relieved by SBC-115076 treatment (Figures 3E,F). All of these effects indicate that PCSK9 may play a significant role in Hcy-inhibited cholesterol efflux mediated by ABCA1 and ABCG1.

### SBC-115076 Decreased the Plasma Hcy and Lipid Levels and Ameliorated Atherosclerotic Lesions in HHcy ApoE^–/–^ Mice

**Figure 4:**
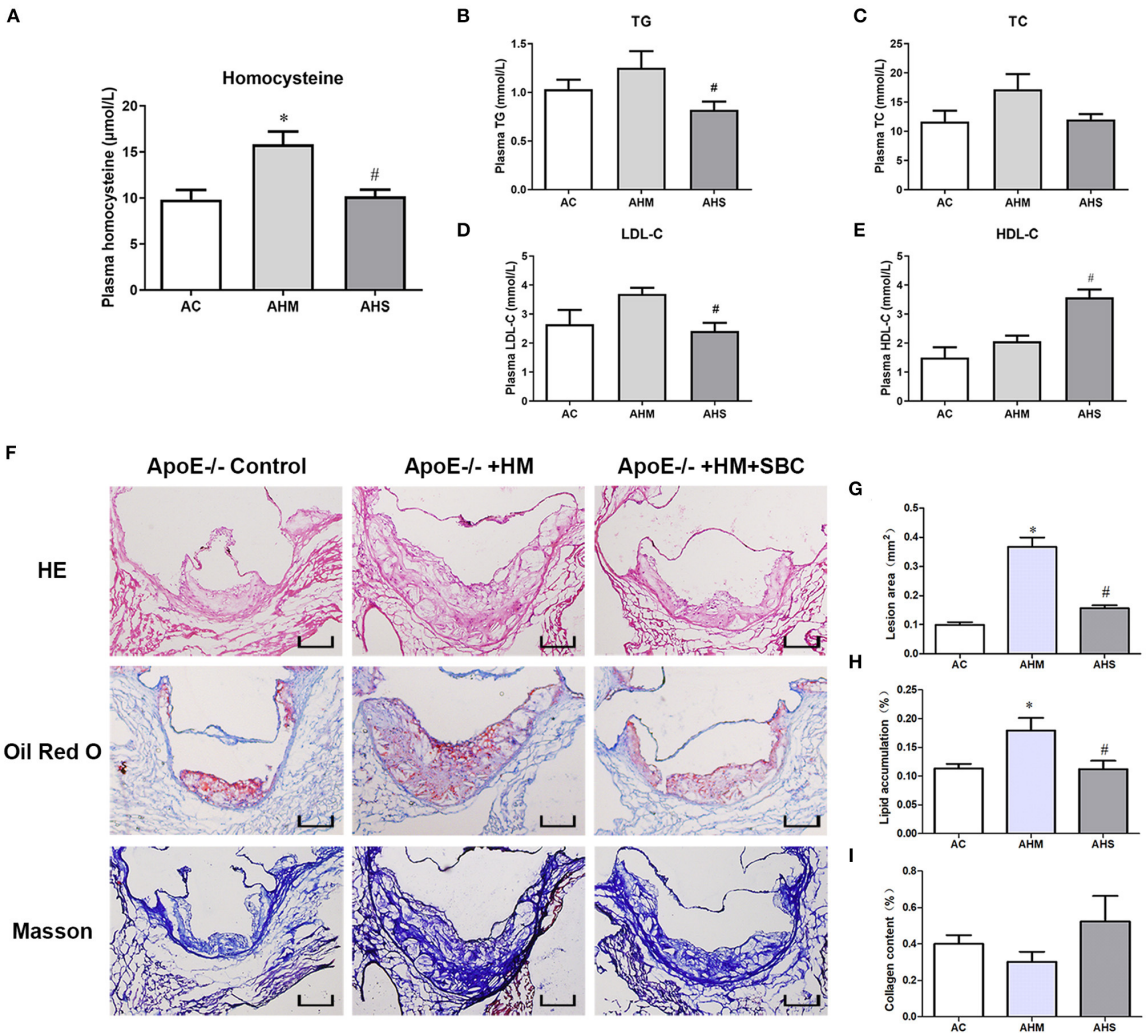
SBC-115076 decreased plasma Hcy and lipid levels and ameliorated atherosclerotic lesions in ApoE^−/−^ mice with HHcy. Levels of plasma Hcy **(A)** and lipid profiles among three groups were measured **(B–E)**. Histology **(F)** and quantification of HE staining **(G)**, Oil-red O staining **(H)** and Masson staining **(I)** in aortic roots. Data represent mean ± standard error of the mean (SEM) (*n* = 12 in each group). *p < 0.05 vs. control group (AC group). ^#^*p* < 0.05 vs. HHcy group (AHM group). Hcy, homocysteine; HHcy, hyperhomocyteniemia; HM, methionine; SBC, SBC-115076; TG, triglycerides; TC, total cholesterol; LDL-C, low-density lipoprotein cholesterol; HDL-C, high-density lipoprotein cholesterol; AC, ApoE^−/−^ mice with chow diet; AHM, ApoE^−/−^ mice with methionine treatment; AHS, ApoE^−/−^ mice with methionine + SBC treatment. Scale bar = 100 μm.

To explore the role of PCSK9 in HHcy-accelerated atherosclerotic lesions *in vivo*, we first fed ApoE^−/−^ mice with a methionine diet for 18 weeks to build a HHcy model (AHM group), as we described previously (10, 21). Then SBC-115076 8 mg/kg was subcutaneously injected (AHS group) to inhibit PCSK9 level *in vivo*. After the intervention, we examined the level of Hcy and lipids profiles in mouse plasma. The HHcy model in the AHM group was successfully established, with plasma Hcy level obviously higher than the control group (AC group) (*p* < 0.05) (Figure 4A). SBC-115076 decreased plasma Hcy level about 40% in AHS group as compared with AHM group. Lipid profiles seemed higher in the HHcy group than control group, but it was not statistically significant (*p* > 0.05) (Figures 4B–E). SBC-115076 significantly decreased triglyceride and LDL-C levels and increased HDL-C levels in the AHS group versus AHM group (*p* < 0.05).

To comprehensively evaluate the effect of SBC-115076 on atherosclerotic lesions in the aortic sinus, we used HE staining to assess lesion area, Oil-red O staining to assess lipid accumulation and Masson trichrome staining to assess collagen deposition in the aortic sinus. Lesion area was increased in the AHM group as compared with the control group, and SBC-115076 treatment in the AHS group greatly reduced the lesion area about 64% as compared with the AHM group (Figures 4F,G). Lipid accumulation in atherosclerotic lesions was enriched in the AHM group, and SBC-115076 treatment in the AHS group reduced lipid deposition as compared with the AHM group. However, there was no statistical difference in collagen deposition among the three groups (*p* > 0.05).

### SBC-115076 Reversed HHcy-Decreased Levels of ABCA1 and ABCG1 in Macrophages From Aortic Lesions in ApoE^–/–^ Mice

**Figure 5:**
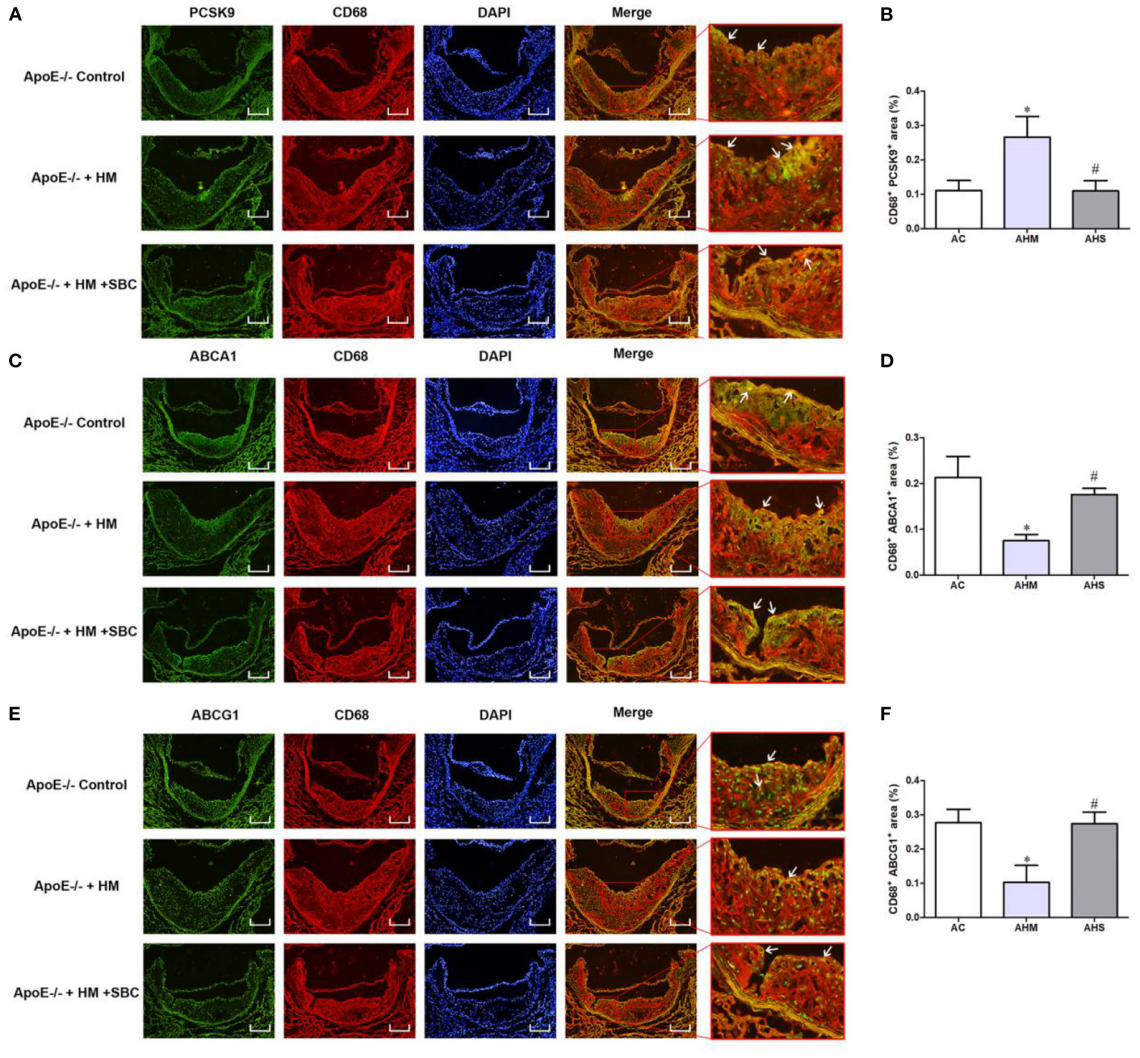
SBC-115076 Reversed HHcy-decreased Levels of ABCA1 and ABCG1 in Macrophages from Aortic Lesions in ApoE^−/−^ Mice. Immunofluorescent double staining and quantification of CD68 antibody and antibodies of PCSK9 **(A,B)**, ABCA1 **(C,D)** and ABCG1 **(E,F)** (white arrows pointed). Green indicated CD68, red indicated PCSK9, ABCA1 and ABCG1, blue indicated DAPI-stained cellular nuclei and yellow indicated double-positive macrophages with 16 × Magnification. Data represent mean ± standard error of the mean (SEM) (*n* = 12 in each group). *p < 0.05 vs. control group (AC group). ^#^*p* < 0.05 vs. HHcy group (AHM group). Hcy, homocysteine; HHcy, hyperhomocyteniemia; HM, methionine; SBC, SBC-115076; AC, ApoE^−/−^ mice with chow diet; AHM, ApoE^−/−^ mice with methionine treatment; AHS, ApoE^−/−^ mice with methionine + SBC treatment. Scale bar = 100 μm.

Because of the potential regulation of PCSK9 of the expression of ABCA1 and ABCG1 in macrophages induced by Hcy mentioned above, we performed immunofluorescence analysis to examine the protein levels of PCSK9, ABCA1 and ABCG1 specifically expressed in macrophages from atherosclerotic plaque. PCSK9 level was increased in macrophages in plaque from the HHcy ApoE^−/−^ mouse model induced with a methionine diet, and SBC-115076 decreased PCSK9 level (Figures 5A,B). Consistent with the results *in vitro*, ABCA1 and ABCG1 specifically expressed on macrophages were downregulated in the AHM group and upregulated in the AHS group about 1.57- and 1.82-fold, respectively (Figures 5C–F).

## Discussion

Since McCully proposed his initial Hcy theory of atherosclerosis in 1969 (25), there has been emerging evidence that Hcy can accelerate the progress of atherogenesis, but the underlying mechanisms are still not completely clarified. In this study, we found that Hcy dose- and time- dependently increased PCSK9 level without decreasing LDLR level in THP-1 macrophages. Furthermore, Hcy inhibited ABCA1- and ABCG1-dependent cholesterol efflux from macrophages mediated by PCSK9, which resulted in cellular lipid accumulation and foam-cell formation. Moreover, in ApoE^−/−^ mice, HHcy amplified atherosclerotic lesion area and aortic lipid deposition by activating PCSK9; inhibiting PCSK9 alleviated atherosclerotic lesion severity and reduced plasma Hcy level and lipid profiles.

Atherosclerosis is a common and basic pathology of many atherosclerotic cardiovascular diseases (ASCVDs), characterized by dyslipidemia, lipid accumulation in intima artery, chronic inflammation and atherosclerotic plaque formation (26, 27). Elevated plasma LDL-C level is a particularly important pathogenic factor of atherosclerosis (28). Deposition of oxidized or acetylated LDL-C and inflammation in macrophages leads to increased plaque instability, accelerated plaque rupture and thrombosis and thus the occurrence of serious clinical cardiovascular events such as myocardial infarction (29). Numerous basic and clinical studies have shown that Hcy can also increase the plaque instability (8, 9, 30). Our previous study (10) showed that HHcy accelerated lipid accumulation and atherosclerotic severity by inhibiting LXRα-ABCA1 and ABCG1 pathway mediated cholesterol efflux. However, although the LXRα agonist T0901317 could reduce the severity of atherosclerotic lesions in part, it could not reduce LDL-C level or even increase triglyceride level. At this time, the timely emergence of PCSK9 and its inhibitors provides a new avenue for this research bottleneck.

PCSK9 is a regulatory protein of lipid metabolism that was discovered by Seidah et al. in 2003 (31). Its pathogenesis in atherosclerosis and application of inhibitors have been widely studied in recent years (14). Most studies of PCSK9 focused on hepatocytes, promoting LDLR degradation in lysosomes by binding with LDLR, thus increasing plasma LDL-C level. Many large clinical trials have revealed that PCSK9 inhibitors can decrease LDL-C level significantly and ameliorate the main adverse cardiovascular events and even reverse plaque accumulation (13, 32, 33). Recent studies have provided more evidence of the PCSK9 on macrophages by promoting cholesterol uptake, inhibiting cholesterol efflux, and activating inflammation or apoptosis (15, 34). We have found (10) that Hcy promoted lipid accumulation mainly by regulating receptors responsible for cholesterol efflux. However, little is known about the role of PCSK9 and its inhibition in Hcy-induced atherosclerosis.

Here, we treated THP-1 macrophages with Hcy and assessed the levels of PCSK9 and LDLR. Hcy dose- and time- dependently increased PCSK9 level, so Hcy can activate PCSK9 expression in macrophages. However, Hcy decreased LDLR mRNA expression without synchronous down-regulation of protein expression, so transcriptional and translational regulation of Hcy on LDLR is not consistent. Recent studies showed that PCSK9 released from smooth muscle cells directly reduced the LDLR expression on macrophages (35) and monocytes (36), which is of vital importance in foam cell formation and atherogenesis. Tang et al. (37) found that PCSK9 can be expressed in THP-1 macrophages and PCSK9 siRNA could suppress inflammation induced by ox-LDL. Our results also showed that PCSK9 can be expressed in macrophages. The above results suggest that the effect of Hcy on PCSK9 in macrophages may not be the same as the pathway of PCSK9/LDLR lysosomal degradation, for which further researches about Hcy on LDLR is needed.

Secondly, we used SBC-115076, a specific antagonist of PCSK9, to verify the effect of PCSK9 in Hcy-accelerated lipid accumulation in macrophages. Our data showed that SBC-115076 exerted no effect on expression of LDLR, which indicated that regulation of SBC-115076 on PCSK9 may have some non-LDLR targets, including mediators of inflammation and immunological processes, such as Apolipoprotein E receptor 2 (ApoER2), very low-density lipoprotein receptors (VLDL-R), LDL-R-related protein 1 (LRP1), CD36, ABCA1, etc. (38). However, SBC-115076 could inhibit the Hcy-induced upregulation of PCSK9. In addition, inhibiting PCSK9 could promote cholesterol efflux to apoA-I and HDL by upregulating the expression of ABCA1 and ABCG1, so inhibiting PCSK9 could relieve the effect of Hcy. However, although Hcy had a significant effect on the reduction of ABCA1 and ABCG1 protein levels, the inhibition of cholesterol efflux to apoA-I and HDL by Hcy was modest. As foam cell formation was determined by uncontrolled cholesterol uptake, excessive cholesterol esterification and impaired cholesterol release. Cholesterol uptake refers to extracellular modified LDL ingested by macrophages via receptors mediated phagocytosis and macropinocytosis (39). SR-A and CD36 have been considered as important receptors responsible for the modified lipoproteins uptake by macrophages. Thampi et al. (40) found that CD36 was upregulated with Hcy activated endothelial cells in peritoneal macrophages. We previously (10) found that Hcy exerted no effect on the expression of CD36, which indicated that effects of Hcy on cholesterol uptake might depend on different cell lines or scavenger receptors. Ding et al. (17) found that PCSK9 regulated the expression of scavenger receptors such as LOX-1 and CD36, then promoted oxidized-LDL uptake in macrophages. Zimetti et al. (41) found that berberine, a known inhibitor of PCSK9, reduced cholesterol uptake by suppressing macropinocytosis and reducing inflammation. Whether CD36 and macropinocytosis is under control of PCSK9 in Hcy-promoted lipid accumulation in macrophages needs verification in more cell lines.

Except the regulation of ABCA1 and ABCG1 on cholesterol efflux, scavenger receptor-BI(SR-BI) also promotes cholesterol efflux from macrophages to HDL. Guo et al. (20) found that SR-BI expression was decreased in both atherosclerotic plaques from ApoE^−/−^ mice with HHcy and Hcy-treated foam cells, and overexpression of SR-BI could abrogate Hcy-induced lipid accumulation. These results indicated that the inhibition of Hcy on cholesterol efflux might be mediated several scavenger receptors, which needs further investigation. Adorni et al. (18) found that PCSK9 plays a direct role in ABCA1-mediated cholesterol by downregulating ABCA1 in macrophages. Inhibition of sterol regulatory element-binding protein (SREBP) expression in the liver has been shown to reduce the levels of PCSK9. Jia et al. (42) found that microRNA-33, an intronic miRNA residing in the SREBP-2 gene, serves a crucial role in the regulation of HDL metabolism, cholesterol efflux and fatty acid β-oxidation through the modulation of the expression of the ABCA1 and ABCG1. It is known that ABCA1 and ABCG1 are target genes of LXRα. Supplementary Material in the present study showed that SBC-115076 put no effect on the expression of LXRα in THP-1 macrophages with or without Hcy. When incubated with T0901317 to activate LXRα, PCSK9 protein expression was decreased. The present results suggested that ABCA1 and ABCG1 might be the downstream target genes regulated by PCSK9 in Hcy-related dyslipidemia of macrophages, and ABCG1 is a new target in the Hcy-activated PCSK9 pathway. And Hcy might affect macrophage cholesterol metabolism by inhibiting the ABCA1 and ABCG1-mediated cholesterol efflux by activating LXR/RXR (18). However, the regulatory relationship between LXRα and PCSK9 was unclear, there might have some interaction between the two, or post-transcriptional regulation of PCSK9, and further researches are needed. If we can inhibit PCSK9 accurately under the condition of Hcy-related cholesterol efflux, then Hcy-related severity of atherosclerotic lesions might be alleviated simultaneously.

Third, we used ApoE^−/−^ mice fed a methionine diet to clarify the role of PCSK9 in atherosclerosis accelerated by Hcy. The HHcy mouse model with a methionine diet was successfully established, with plasma Hcy levels increased significantly. We observed a higher lipid level profile in the HHcy group(AHM) but with no significant difference as compared with the control group (AC). With SBC-115076 subcutaneous injection (AHS) to inhibit PCSK9, lipid profiles were significantly reduced as compared with the HHcy group (AHM). Thus, this intensive lipid-lowering effect of PCSK9 inhibition is still remarkable and stable in HHcy-related atherosclerosis. In addition, inhibiting PCSK9 also reduced plasma Hcy level. As we just used only one inhibitor SBC-115076 and found Hcy level was reduced after SBC-115076 intervened. We thought there might be some components in SBC-115076 that could itself influence Hcy level; or inhibition of PCSK9 triggered the metabolism of Hcy *in vivo*. Therefore, it was difficult to accurately explain the relationship between Hcy and PCSK9.

With histochemical staining, we found significantly increased lesion area and lipid accumulation of aortic plaques in the HHcy group (AHM); SBC-115076 treatment (AHS) reduced the lesion area and lipid accumulation. Thus inhibiting PCSK9 could alleviate HHcy-related severity of atherosclerotic lesions. However, we did not evaluate the stability of plaque with lipid accumulation, collagen deposition, macrophage infiltration or smooth muscle cell proliferation among the three mouse groups. We know that Hcy is a strong residual risk factor after intensive statin therapy among ASCVD patients, and even if using folic acid and vitamin B12 to reduce plasma Hcy level, the improvement in the main adverse cardiovascular outcomes is still limited (43). The above results suggest that if PCSK9 can be used as a potential intervention target. The lipid profiles, plaque stability and prognosis of HHcy-related ASCVD can be improved, which shed light on the clinical treatment of patients with HHcy-related ASCVD.

Although the present study proposes a new mechanism for the important role of PCSK9 in Hcy-induced lipid accumulation in macrophages and the acceleration of atherosclerosis, there are still some limitations in this study. First, we just used only one antagonist SBC-115076 to inhibit PCSK9 *in vitro* and *in vivo* study, further researches using more antagonists, monoclonal antibodies or even gene knock out models of PCSK9 are needed to comprehensively clarify the effects of PCSK9. Second, we did not have a bland control group for SBC-115076 *in vivo* study, which suggested that we should be more comprehensive and objective in grouping *in vivo* experiments. Meanwhile, we did not provide an assessment of the stability of aortic plaque to further clarify the stabilizing plaque effect of inhibiting PCSK9. Therefore, more in-depth and complex research is needed to thoroughly clarify the mechanism of PCSK9 in Hcy-induced atherosclerosis, to provide more professional evidence for clinical intervention in patients with HHcy-related ASCVD. Third, regulation of PCSK9 on ABCA1 and ABCG1 should be researched based on activation of LXR/RXR, so as to have a better understanding of PCSK9 and LXR-ABCA1/ABCG1 signaling pathway in condition of Hcy.

In conclusion, the present study found that Hcy inhibited ABCA1 and ABCG1-dependent cholesterol efflux by activating PCSK9, thereby promoting lipid accumulation in macrophages and accelerating the severity of atherosclerotic lesions. Inhibiting PCSK9 significantly reduced the plasma Hcy level, altered lipid profiles and reduced the severity of atherosclerotic lesions caused by HHcy. These findings provide novel insights into the pathogenesis of Hcy-induced atherosclerosis. PCSK9 inhibitor treatment may be a promising intervention in the clinic for ASCVD patients with HHcy.

## Data Availability Statement

The original contributions presented in the study are included in the article/Supplementary Material, further inquiries can be directed to the corresponding author/s.

## Ethics Statement

The animal study was reviewed and approved by the committee on the Ethics of Animal Experiments of the Health Science Center of Ningxia Medical University.

## Author Contributions

SJ conceived and designed the experiments. PJ, GC, and RY performed the experiments. PJ and GC analyzed the data. PJ drafted the manuscript. DG polished the article. All authors read and approved the final manuscript.

## Funding

This work was financially supported by grants from the National Natural Science Foundation of China (No. 81660060), the Key Research and Development Projects of Ningxia (No. 2018BEG02006), Foundation of Xi'an Jiaotong University (No. xzy012020102), and Foundation of The Second Affiliated Hospital of Xi'an Jiaotong University (No. YJ(QN)201916).

## Conflict of Interest

The authors declare that the research was conducted in the absence of any commercial or financial relationships that could be construed as a potential conflict of interest.

## Publisher's Note

All claims expressed in this article are solely those of the authors and do not necessarily represent those of their affiliated organizations, or those of the publisher, the editors and the reviewers. Any product that may be evaluated in this article, or claim that may be made by its manufacturer, is not guaranteed or endorsed by the publisher.
