# Supplementary material for: Role of PCSK9 in Homocysteine-Accelerated Lipid Accumulation in Macrophages and Atherosclerosis in ApoE−/− Mice
Source: Front Cardiovasc Med. 2021 Oct 1;8:746989. doi: 10.3389/fcvm.2021.746989 (PMC8517151; doi:10.3389/fcvm.2021.746989)
Supplement: Supplementary file 1 [file Data_Sheet_1.PDF]

Supplemental Materials

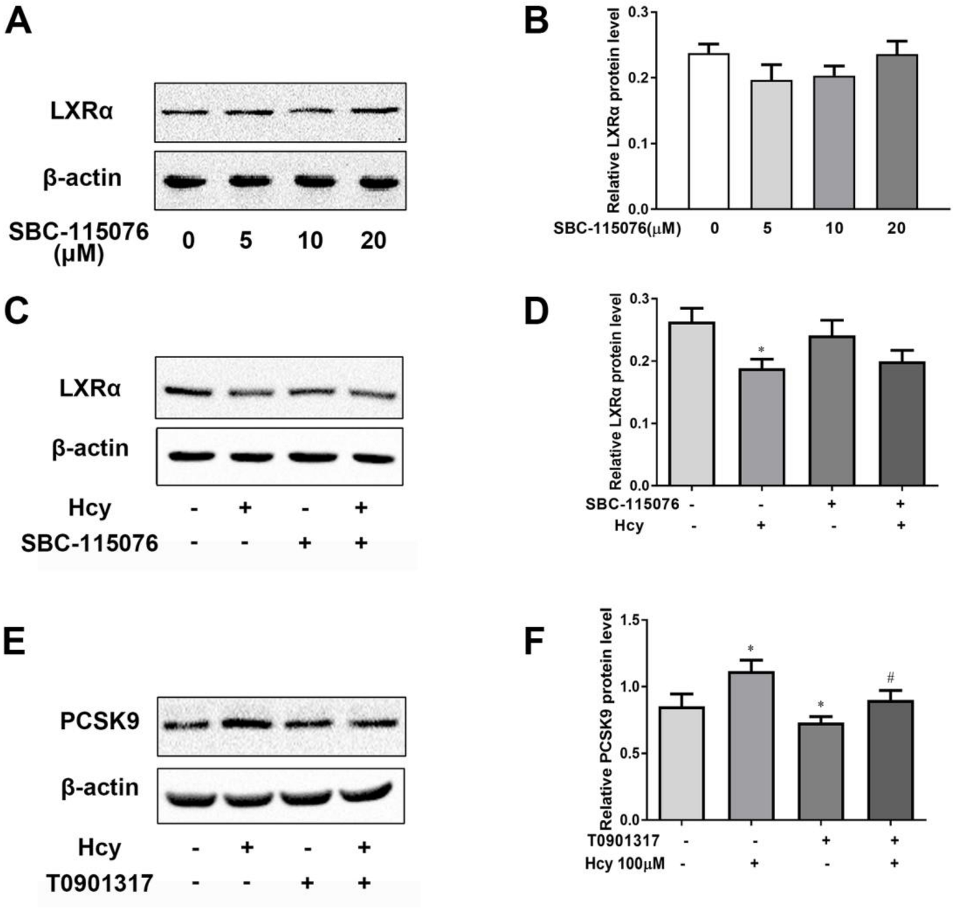

FIGURE SUPPLMETNT 1 | SBC-115076 had no effect on the protein expression of LXRα with or without Hcy intervention. Protein level of LXRα was determined by western blot analysis with 5, 10, 20 μmol/L SBC-115076 for 24h (A, B) in THP-1 macrophages. Then cells were co-incubated with 100μmol/L Hcy and 20μmol/L SBC-115076 for 24h. Protein level of LXRα was determined (C, D). In addition, protein level of PCSK9 was determined by western blot analysis when cells were co-incubated with 100μmol/L Hcy and 5μg/mL T0901317 for 24h(E, F). Data represent mean ± standard error of the mean(SEM). Each experiment was performed three times. *$p < 0.05$ vs. the control group(which refers to incubation with PBS only). #$p < 0.05$ vs. Hcy group(Hcy 100μmol/L). Hcy, homocysteine.
